# Negligible therapeutic impact, false-positives, overdiagnosis and lead-time are the reasons why radiographs bring more harm than benefits in the caries diagnosis of preschool children

**DOI:** 10.1186/s12903-021-01528-w

**Published:** 2021-03-31

**Authors:** Laura Regina A. Pontes, Juan Sebastian Lara, Tatiane Fernandes Novaes, Julia Gomes Freitas, Thais Gimenez, Bruna Lorena P. Moro, Haline C. M. Maia, José Carlos P. Imparato, Mariana M. Braga, Daniela P. Raggio, Fausto M. Mendes, CARDEC collaborative group

**Affiliations:** 1 Department of Pediatric Dentistry, School of Dentistry, Faculty of Dentistry, University of São Paulo, Av. Lineu Prestes, 2227, São Paulo, SP 05508-000 Brazil; 2 Department of Cariology, Operative Dentistry and Dental Public Health, Dental Institute, Indiana University School of Dentistry, 1121 W Michigan St., Indiana, IN 46202 USA; 3 Cruzeiro Do Sul University, R. Galvão Bueno, 868, São Paulo, 01506-000 Brazil; 4 School of Dentistry, Ibirapuera University, Av. Interlagos, 1329, São Paulo, 04661-100 Brazil

**Keywords:** Clinical trial, Dental caries, Radiography, Primary teeth, Diagnosis, Children

## Abstract

**Background:**

To evaluate the clinical course and interventions required during two years of follow-up of dental surfaces of deciduous molars diagnosed, and consequently treated, by two different strategies: diagnosis made by clinical examination alone or associated with radiographs.

**Methods:**

This is a secondary analysis of a two-arm randomized clinical trial with parallel groups related to the diagnostic strategy for caries detection in preschool children. 216 children (3–6 years old) were followed-up for two years. All dental surfaces were diagnosed by visual inspection and later, through radiographic assessment. Baseline treatment was made in accordance with the results obtained by visual inspection performed alone or combined with radiographic method, considering the allocated group. Dental surfaces with no restoration needs, or those restored at the beginning of the study were followed-up for two years. The treatment decision was made according to the allocated group. The outcome was the occurrence of failure (a new caries lesion or a restoration replacement) during the follow-up.

**Results:**

4383 proximal and occlusal surfaces of deciduous molars in 216 preschool children were diagnosed and treated according to the abovementioned diagnostic strategies and followed-up for 24 months. The assessment of radiographs made change the initial decision reached by visual inspection in about 30% of the surfaces when all types of interventions were considered. However, most disagreements occurred for initial lesions, where radiographs tended to underestimate them. Discordances between methods occurred in less than 5% of all surfaces when considered lesions requiring operative treatment. For discrepancy cases, the placed interventions guided by following the radiographic results did not present less failures against those made following only visual inspection. As a matter of fact, the use of radiographs in the diagnostic strategy for caries detection in children brought more harms than benefits due to the occurrence of false-positives, overdiagnosis and lead-time bias.

**Conclusions:**

Simultaneous association of visual inspection and radiographic assessment for caries detection in preschool children causes more harms than benefits, and therefore, visual inspection should be conducted alone in the regular clinical practice.

*Trial registration* Clinicaltrials.gov platform: NCT02078453, registered on 5th March 2014.

**Supplementary Information:**

The online version contains supplementary material available at 10.1186/s12903-021-01528-w.

## Background

The current paradigm related to diagnosis in health care is the early, even presymptomatic, detection of diseases [1–4]. In this way, asymptomatic people are encouraged to attend at regular health checkups in order to keep their well-being [2].

Nevertheless, this movement of making more and earlier diagnosis has a side effect: many people are considered sick, even though these diseases would not actually cause any problem during their lifetime [5]. This is defined as overdiagnosis; subjects that are diagnosed with conditions that neither would cause any symptoms and harms, nor would be the cause of death of those affected [5–7]. This issue has been extensively investigated in adults’ health care [7], but the occurrence of overdiagnosis has also been raising awareness in Pediatrics [6, 8].

The same trend can be observed in dental practice. People are advised to visit the dentist at recall intervals varying from 3 to 12 months for children and 3–24 months for adults [9–11], even though the effectiveness and ideal interval of regular checkups are still unclear [10]. Moreover, early diagnosis of different oral health-related conditions has been proposed [12, 13], especially for dental caries [9, 14–17].

Dental caries, also known as dental decay, is a non-communicable disease mediated by the biofilm formed on dental surfaces and modulated by the diet, mainly fermentable carbohydrates. It is a dynamic process consisting of alternating periods of demineralization and remineralization. When the net mineral loss is predominant during a period, an initial caries lesion becomes clinically detectable. With the progression of the disease, caries lesions of different stages can affect children, adults and elderly from initial lesions restricted to the enamel to deep cavities exposing the pulp [15, 18, 19]. Dental caries is the most prevalent oral health condition [20] and consistently causes a negative impact on the quality of life in all age groups [21]. As regards childhood, untreated dental caries in deciduous teeth affects around 500 million children, being the most prevalent chronic disease at this age group [22].

Currently, the diagnostic strategy for caries lesions detection indicated in most clinical guidelines is the clinical examination simultaneously associated with a radiographic assessment [9, 17, 23–26]. Visual inspection must be performed in all patients at the beginning of the treatment, and the method presents high specificity for the detection of caries lesions. However, clinical examination tends to overlook several caries lesions requiring operative treatments, mainly at occlusal and proximal surfaces of posterior teeth [26, 27].

Given this low sensitivity, radiographs have been indicated as an adjunct method to associate with clinical examination. The first argument for such recommendation is that the assessment of radiographs increases the sensitivity of the visual inspection used alone. Therefore, many lesions requiring operative interventions (at most advanced stages) and missed during clinical examination, could be detected [17, 23, 26]. Another advantage would be the early detection of caries lesions before a cavitation is present, and therefore, treated them non-operatively, avoiding a more invasive approach [9, 17, 24–26]. In both situations, a simultaneous diagnostic strategy is advocated. This combination of visual inspection with radiographic methods, however, has been challenged by studies conducted in representative samples, mainly for the detection of caries lesions in deciduous teeth [28, 29].

Another possibility is the use of radiographs in a sequential combination with visual inspection [29–31]. In this way, the method would be used to confirm a positive result obtained with the visual inspection, increasing the certainty on the actual necessity of operative treatment. However, the utility of using a radiographic assessment in the caries diagnosis strategy has been tested only with accuracy studies, most of them with high risk of selection bias [26, 27]. No previous research has investigated the benefits of caries detection methods evaluating patient-centered outcomes through a randomized clinical trial. Therefore, the use of radiographs for caries detection, mainly in children, is controversial.

The problems with this tendency of early detection of many diseases is that although a very small number of patients would benefit from the early detection of some life-threatening diseases, many others would suffer anxiety and adverse effects of the unnecessary treatment of a problem that would never bother them [6–8, 32, 33]. Additionally, this excess of diagnosis has an obvious economic impact for most stakeholders [5–7, 33]. Thus, diagnostic strategies for prevalent and disabling diseases, such as dental caries, should be tested through randomized clinical trials in order to strengthen the evidence on their use in daily clinical practice.

Considering this scenario, we pioneered the conduction of clinical trials on caries diagnosis strategies used in children. The CARies DEtection in Children (CARDEC) trials initiative began with a randomized clinical trial testing two diagnostic strategies (and subsequent treatment) for caries detection in deciduous teeth of preschool children: visual inspection performed alone or simultaneously combined with a radiographic assessment. The main trial compared participants for the occurrence of new operative interventions during a two-year follow-up [34, 35].

The present study, containing secondary analyses of data from the main trial, aimed to evaluate the placed interventions and clinical course on occlusal and proximal surfaces of deciduous molars of children diagnosed by both visual inspection and in combination with the radiographic method after 2 years. The specific aims were to compare the two diagnostic strategies regarding the therapeutic impact, proportion of false-positives and overdiagnosis, and occurrence of lead-time bias. For these analyses, the dental surfaces of primary molars were the unit of analysis, and we evaluated the clinical course of surfaces that were diagnosed as sound or decayed at the baseline. Treatment decision was performed according to the allocated diagnostic strategy. The comparison of the results obtained with the different diagnostic methods was used to assess the therapeutic impact and false-positive results and the clinical course of the treatment performed was important to assess occurrence of overdiagnosis and lead-time.

## Material and methods

### Trial design

The present study is a secondary analysis from a randomized clinical trial carried out to investigate diagnostic strategies for caries detection in preschool children. The main clinical trial, entitled CARies DEtection in Children-1 (CARDEC-01), had its protocol approved by the Research Ethics Committee of the School of Dentistry, University of São Paulo (CAAE number 02952612.4.0000.0075), and it was registered on the ClinicalTrials.gov platform (NCT02078453). Moreover, the protocol was previously published [34].

In short, the CARDEC-01 study is a two-arm, randomized, parallel design clinical trial with two years of follow-up comparing two different diagnostic strategies for caries detection in children aged from 3 to 6 years: caries lesion detection and subsequent treatment conducted using only visual inspection (VIS group), and caries lesion detection and dental treatment performed through the simultaneous association of visual inspection and radiographic method (RAD group). For the latter strategy, a positive result obtained with one of the methods would classify the surface as decayed. The primary endpoint was the number of new operative interventions performed on deciduous molars during the two-year follow-up and our findings were recently published [35]. Data related to oral health-related quality of life and economic analysis will be further analyzed and published separately.

In the present study, secondary analyses were performed considering the dental surface of deciduous molars as the unit of analysis. Dental surfaces were clustered on deciduous molars, which were clustered in the children. This 3-level cluster structure was considered for all analyses. These analyses have not been predetermined in the protocol [34].

### Participants

Children from 3 to 6 years old, whose parents looked for dental treatment in the School of Dentistry of the University of São Paulo, São Paulo, Brazil, were eligible to participate. Children, whose parents did not agree with the participation, did not assent to take part in the study or offered difficulties in the behavior management during the first dental appointments, were excluded. A researcher (LRAP), graduate student of Pediatric Dentistry, responsible for participants’ enrolment, conducted initial dental examinations and took two bitewings in all included children. Bitewings, also known as coronal radiographs or proximal radiographs, are taken to visualize the crowns of posterior teeth, and are commonly indicated for caries detection. Other periapical radiographs were also taken, when necessary. All procedures, including diagnosis and subsequent dental treatments, were conducted at the dental office setting.

### Interventions

All interventions were performed in accordance with the diagnostic strategies to which participants were allocated. Therefore, and as described above, two trial groups—VIS and RAD—constituted this study.

One of two trained and calibrated examiners (JSL and TFN) conducted the diagnostic procedures. The examiners were graduate students (PhD) in Pediatric Dentistry. Examiners obtained inter and intra-examiner weighted kappa values higher than 0.80 before the beginning of the study for both visual inspection and radiographic methods. Details on the calibration procedures can be assessed elsewhere [34]. Assessments were carried out in a dental chair under artificial light. Teeth were previously cleaned with rotating bristle brush, pumice/water slurry and dental floss. Teeth were examined with the aid of a plane buccal mirror and a ball-point probe. The examiners visually assessed each dental surface, first wet, and then, air-dried for 5 s, classifying findings according to the International Caries Detection and Assessment System (ICDAS) associated with lesion activity assessment [36, 37].

The radiographic method was conducted evaluating two bitewings, taken with 22X35mm films (Kodak Insight, Eastman Kodak, Rochester, USA) in an X-ray machine (Spectro X70, Dabi Atlante, Ribeirão Preto, Brazil) set at 70 kV and 8 mA. Films were manually developed, and the examiners evaluated images in the backlit screen with no magnification.

VIS group children received an examination through visual inspection, and their treatment plan was created based exclusively on this evaluation. No specific treatment was decided for dental surfaces of deciduous molars classified as sound (score 0 of the ICDAS). The same decision was made for the inactive caries lesions. For active caries lesions ICDAS scores 1–3, a decision of non-operative treatment was reached. Moreover, lesions classified as ICDAS scores 4–6 were indicated for operative treatment. After planning dental treatments by visual inspection, the bitewings were disclosed to the examiners, and then, they classified the proximal and occlusal surfaces of deciduous molars according to the radiographic images, elaborating a new treatment plan. The dental interventions, however, were performed considering the first management plan. Differences between the two treatment plans created for the VIS group children were analyzed in a before-after study, published elsewhere [38].

In the RAD group, the examiners received the bitewings before the clinical examination, and the evaluation of both methods was used for caries diagnosis and treatment plan decision. As a simultaneous strategy was used, a positive result in any method would be sufficient to classify the surface as decayed. In relation to radiographic images, the absence of radiolucencies led to a decision of no treatment necessary. Dental surfaces with radiolucencies restricted to enamel were indicated for non-operative treatment, and surfaces with radiolucencies reaching the dentin were indicated for operative treatment. In case of discordances between both methods, the most severe classification was considered.

Surfaces with no treatment needs did not receive any specific treatment. Non-operative treatment in our study was performed by applying a 22,600 ppm fluoride varnish (Duraphat, Colgate-Palmolive, Waltrop, Germany) on the caries lesion. Operative treatment was conducted with selective caries removal and restoration using high-viscosity glass ionomer cement (Fuji 9 Gold Label, GC Corp., Leuven, Belgium). Regardless of the allocation group, all children received dietary and oral hygiene instructions including the use of fluoridated toothpaste as standard care for dental caries. Detailed treatment protocols were described in a previous publication [34].

### Outcomes

The primary and secondary outcomes of the main trial were variables related to children [34, 35]. In the present study, surfaces with previously placed restorations, or teeth submitted to endodontic treatment or extraction were excluded from the analysis. Therefore, this secondary analysis focused on only those deciduous molars surfaces that did not receive operative intervention or that were restored at the beginning of the study. Moreover, we considered only proximal and occlusal surfaces of deciduous molars.

Therefore, a failure in the present study was considered as the occurrence of a new operative intervention in the abovementioned surfaces, placed within the follow-up period. All events representing the outcome variable were evaluated during the follow-up and could be: (1) a new restoration due to a new caries lesion, (2) a restoration replacement, (3) a tooth surface with endodontic treatment indication or (4) extraction indication.

A new restoration was indicated for dental surfaces with ICDAS scores 5 or 6 [37]. The decision for replacing restorations was based on two criteria previously published [39, 40]. Restorations with marginal defects deeper than 0.5 mm, with some breakdown, with dentin caries around or totally missed were replaced.

Children were scheduled to return every 6 months after the end of the dental treatment for two years. Parents were instructed to return if any treatment need was perceived. The outcome was assessed by an independent assessor (DPR).

### Sample size, randomization, and blinding

Sample size was calculated considering the primary outcome of the main trial, and the minimum sample size calculation estimated the number of 250, anticipating an attrition rate of 20%. Details were previously published [34, 35].

For the randomization, an allocation rate of 1:1 between the groups was used. The random sequence was generated by a researcher who were not involved in the clinical procedures (FMM), using the website www.sealedenvelope.com, in blocks of eight numbers, stratified by children’s age (3 and 4, or 5 and 6 years old) and caries experience (number of surfaces from deciduous teeth decayed, missed or filled—dmf-s from 0 to 3, or children with dmf-s > 3).

The sequence generated was closed in opaque envelopes numbered sequentially according to the strata. Envelopes were opened only after dental prophylaxis, with children positioned on the dental chair and prior to the diagnostic assessment. As stated before, bitewings were taken from all children. However, they were initially disclosed to the examiners only for children allocated to RAD group.

This study was triple-blind. Children and parents were blind to the allocation groups. Furthermore, the dentists that performed the dental treatments according to the pre-determined treatment plans (care providers) and the outcome assessor were also blinded in relation to the diagnostic strategy used in each child. Contrariwise, the researcher responsible for participants’ enrolment and the examiners were not blinded.

### Data analyses

We built a decision tree representing the treatment plan using visual inspection and then, the radiographic method. Subsequently, and in the same tree, we represented the treatment that was actually performed for each possible combination of results, and the outcome (success or failure) after the follow-up. A failure was considered as the necessity of a new operative intervention within the follow-up period. This decision tree permitted the evaluation of the therapeutic impact of different associations between visual and radiographic methods used for caries detection in the deciduous molars of the children, considering all possibilities of interventions. In the first decision tree, we recorded all possible treatments performed at the beginning of the study: no treatment required, requirement of a non-operative treatment, or requirement of restoration (operative treatment). The actual numbers and frequency values in relation to all dental surfaces included were calculated for each branch in the decision tree. Moreover, the respective 95% confidence interval (95%CI) values of each frequency were calculated using an appropriate approach for the clustered nature of the data [41]. The option for deriving the probabilities in relation to the total number of surfaces was followed since the use of natural frequencies tended to be interpreted more accurately [42], and these figures offer a more real understanding about each diagnostic result impact and subsequent treatment performed.

A new decision tree was drawn, but this time only considering the decision for operative treatments. Also, actual numbers, frequencies in relation to the total of included surfaces and respective 95%CIs adjusted by the cluster were plotted. In this analysis, besides the therapeutic impact of the association of methods in the decision for operative treatment, we also figured out the number of false-positive results obtained with both methods, and estimated the overdiagnosis made by the radiographic method in surfaces classified as sound by the clinical examination. Comparisons between failure rates occurring in specific conditions were established using multilevel logistic regression analysis at three levels: dental surfaces (1st level), deciduous molars (2nd level), and children (3rd level). When using this approach, odds ratio (OR) values and respective 95%CIs were derived. Moreover, this decision tree permitted the evaluation of the frequency of situations (success) that were benefitted by the radiographic method.

Another multilevel logistic regression analysis was conducted to investigate the factors associated with the occurrence of a new operative intervention (outcome variable) during the follow-up. The main exposure variable was the different combination of results obtained with both visual and radiographic methods for the dental surfaces. First, OR values and 95%CIs of all explanatory variables were calculated in univariate analyses.

**Fig. 1:**
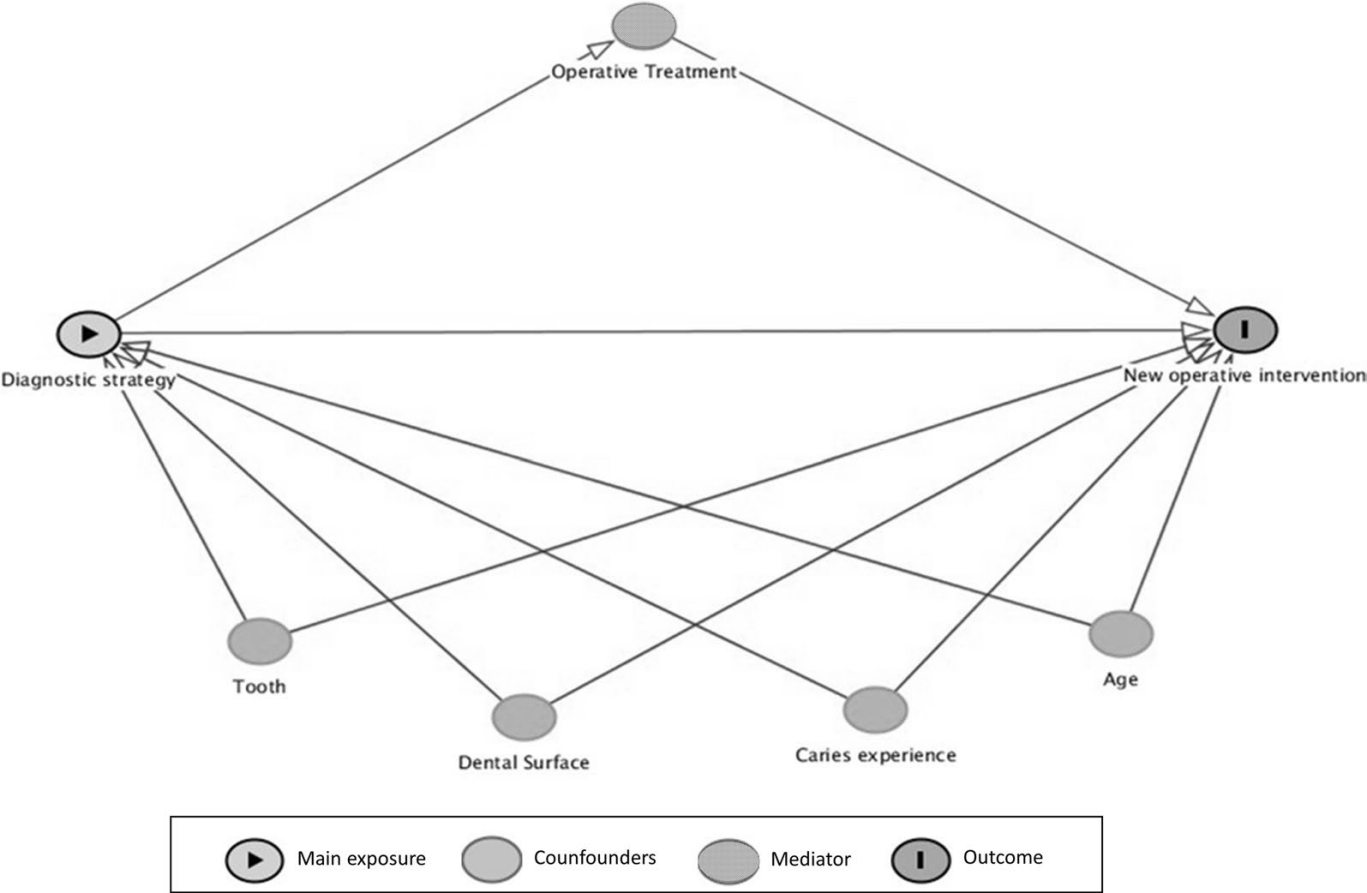
Conceptual framework built to perform the multilevel logistic analysis to evaluate the influence of the results obtained with different diagnostic strategies on the occurrence of new operative treatments during the follow-up

Then, multiple regression analyses were performed following the structure of a conceptual framework previously developed (Fig. 1). This framework included the main explanatory variable (results from the caries diagnostic procedures), the outcome (new interventions during the follow-up), some confounding variables (child’s age, caries experience, type of deciduous molar and type of dental surface) and a possible mediator (performing or not the restoration at the beginning of the study). A first multiple model was built including all confounders; then, a second multiple model was derived adding the mediation variable.

Additionally, we carried out a mediation analysis to evaluate if performing a restoration in dental surfaces with a negative result obtained with the visual inspection, but positive through radiographic evaluation, could exert a mediation effect on the occurrence of failures. In this analysis, regression coefficients and standard errors were derived using multilevel logistic regression analysis, adjusted by the confounding variables. To evaluate the statistical significance of the mediation effect, we used the Sobel test.

Finally, to investigate the possibility of occurrence of lead-time bias, survival analysis considering multiple-failure-time was conducted only in the surfaces diagnosed as negative by visual inspection, but as positive by the radiographic method. According to this situation, children allocated to the RAD group would receive restorations at the beginning of the study, while in the children allocated to VIS group, these surfaces would not be restored at the baseline, would only be treated if an evident caries lesion was noted during the follow-up. Time 0 for each surface was adjusted as the birth date of the children in the survival analysis. Then, the first event was always determined as the first restoration placed on the dental surface. The time when this restoration was placed was also recorded. The subsequent failures were due to restoration failures. The main explanatory variable was the conduction of the restoration at the beginning of the study or not. The method for analysis was Cox regression using conditional risk set model and Efron’s method for handling ties, and the hazard ratio (HR) and the respective 95% CI were calculated. The data was presented in a time-to-event graph.

The conceptual framework was built on the DAGitty website (www.dagitty.net). All statistical analyses were conducted using two statistical packages: Stata 13.0 (Stata corp. College Station, USA) and MedCalc 18.5 (MedCalc software bvba, Ostend, Belgium). The level of significance was set at 5% for all analyses.

## Results

### Characteristics of the participants and dental surfaces included

Initially, 252 children were included from March 19, 2014 to November 25, 2015. From these, 216 were followed-up until 24 months (attrition rate of 14.3%).

One hundred and six children from the visual inspection group and 110 from the radiographic method group finished the study (*p* = 0.108, by chi-square test) in the mail trial. Full data was published elsewhere, including the flow chart and participants’ data at baseline [35].

Since only occlusal and proximal surfaces of deciduous molars that did not receive any type of treatment, or were submitted to non-operative or restorative treatment at the baseline were considered for this study’s purpose, we analyzed 4,383 dental surfaces (66.7% of proximal and 33.3% of occlusal surfaces) in 1461 molars, being 720 first (49.3%) and 741 second (50.7%) deciduous molars of 216 children. One hundred and eight (50.5%) were male and 106 (49.5%) female, 107 (50.0%) were 3 or 4 years old and 107 (50.0%) were 5 or 6 years old. Moreover, 97 (45.3%) children had a dmf-s from 0 to 3, and 117 (54.7%) children presented a dmf-s higher than 3.

### Caries detection and treatment conducted considering all types of treatments

**Fig. 2:**
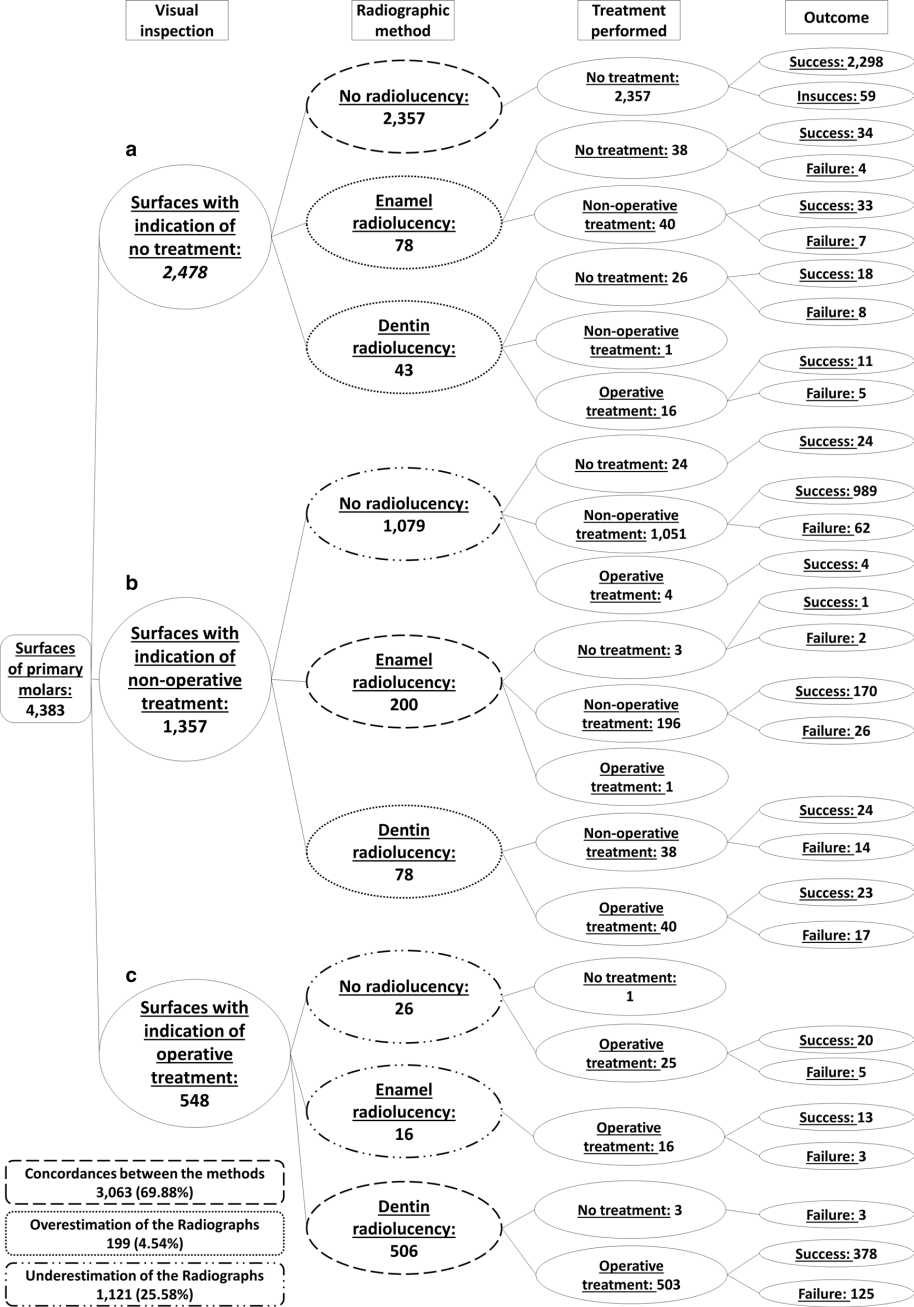
Decision tree (general view) considering caries lesions detection and subsequent treatment (no treatment, non-operative and operative treatment) performed first with visual inspection and then with the adjunct radiographic method in occlusal and proximal surfaces of primary molars. Values indicate the absolute number of surfaces

Figure 2 shows the decision tree with the diagnosis and subsequent dental treatment performed using, firstly, visual inspection alone and then adding the radiographic assessment. Both methods were coincident for most surfaces (almost 70% of all surfaces). Considering sound surfaces, initial and more advanced caries lesions in the assessment, the radiographic method underestimated the diagnosis and treatment decision made by visual inspection in around 25% of the surfaces, and overestimated the treatment decision in 4.5% of the surfaces (Fig. 2). Underestimation occurred mainly in surfaces with initial caries lesions according to the visual inspection (indicative of non-operative treatment), but for which the radiographs did not present any radiolucency (Fig. 2, pathway b).

**Fig. 3:**
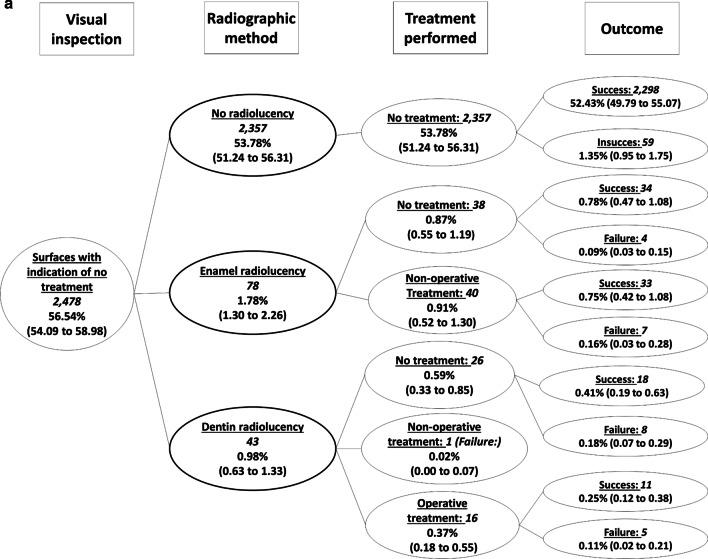
Decision tree focused on the dental surfaces with the indication of no treatment according to the visual inspection (Fig. 2, pathway a). Italicized numbers indicate the number of surfaces. Unformatted numbers indicated the frequency in relation to the total number of surfaces (n = 4383), and figures in parenthesis are the 95% confidence intervals adjusted by the cluster

We found only 121 dental surfaces (less than 3% of all surfaces examined) classified as sound (an indication of non-local treatment) by visual inspection, but with a radiolucency in enamel or dentin in the radiographs (Fig. 2, pathway a, and Fig. 3). This situation has been advocated as one of the advantages of taking bitewings in the clinical practice, since non-operative treatments could be performed to avoid caries lesion progression and cavitation. From these 121 surfaces, non-operative treatment was performed in 41 surfaces, while 64 surfaces did not receive any type of treatment (16 surfaces were restored). Besides the low occurrence of this situation, the frequency of failures (cavitation during the follow-up) of the untreated surfaces was 18.8%, while failures in the surfaces submitted to non-operative treatment occurred in 19.5% (OR = 1.05; 95%CI = 0.35 to 3.09) (Fig. 3).

**Fig. 4:**
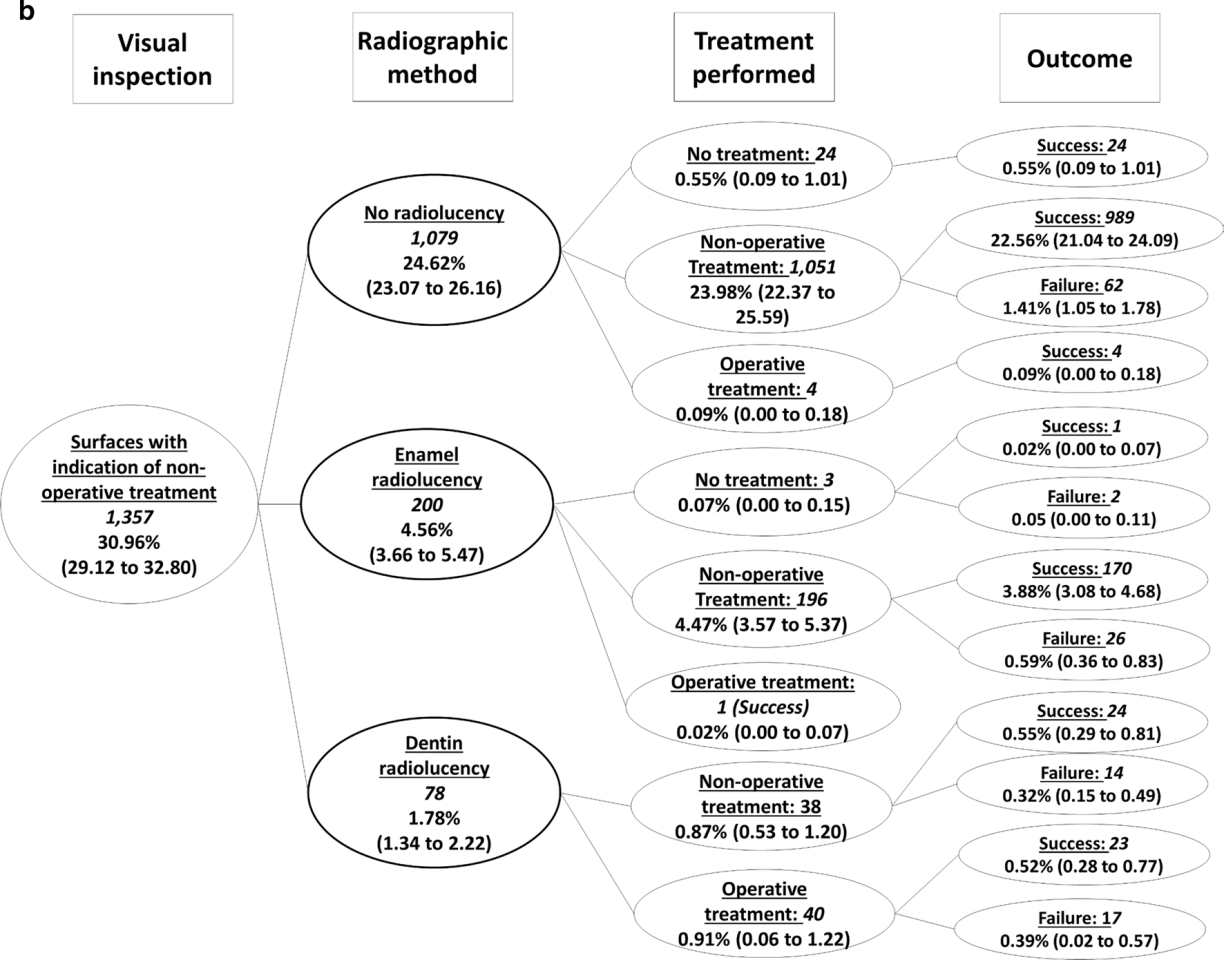
Decision tree focused on the dental surfaces with the indication of non-operative treatment according to the visual inspection (Fig. 2, pathway b). Italicized numbers indicate the number of surfaces. Unformatted numbers indicated the frequency in relation to the total number of surfaces (n = 4383), and figures in parenthesis are the 95% confidence intervals adjusted by the cluster

**Fig. 5:**
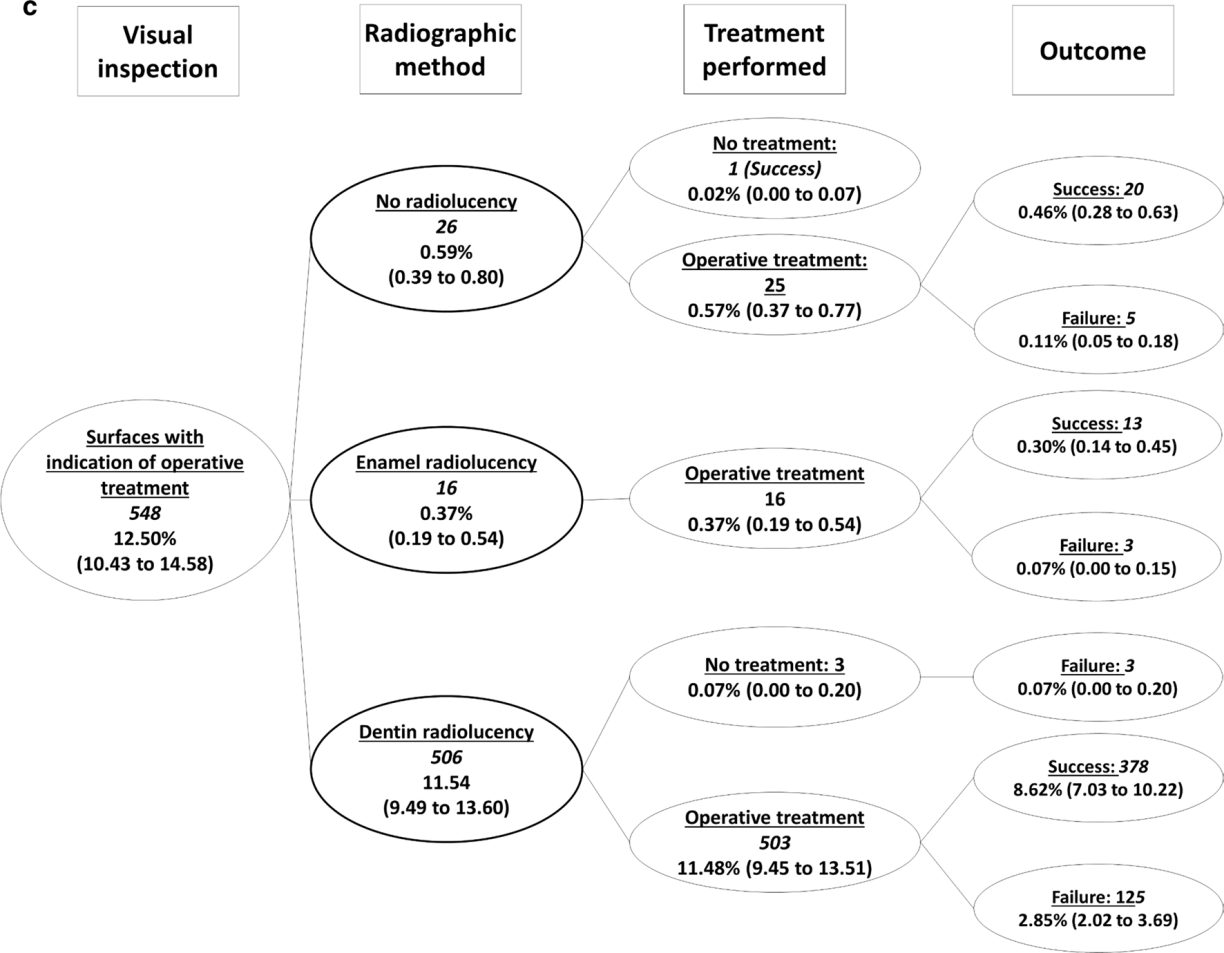
Decision tree focused on the dental surfaces with indication of operative treatment according to the visual inspection (Fig. 2, pathway c). Italicized numbers indicate the number of surfaces. Unformatted numbers indicated the frequency in relation to the total number of surfaces (n = 4383), and figures in parenthesis are the 95% confidence intervals adjusted by the cluster

From the 1357 surfaces presenting initial caries lesions detected by visual inspection, radiographs did not show radiolucencies in 1079 dental surfaces (Fig. 4). Extensive caries lesions detected by visual inspection were observed in 548 dental surfaces, and from these, only in 42 surfaces the radiographic examination did not present radiolucencies reaching the dentin (Fig. 5).

### Caries detection related to the decision for operative treatment

**Fig. 6:**
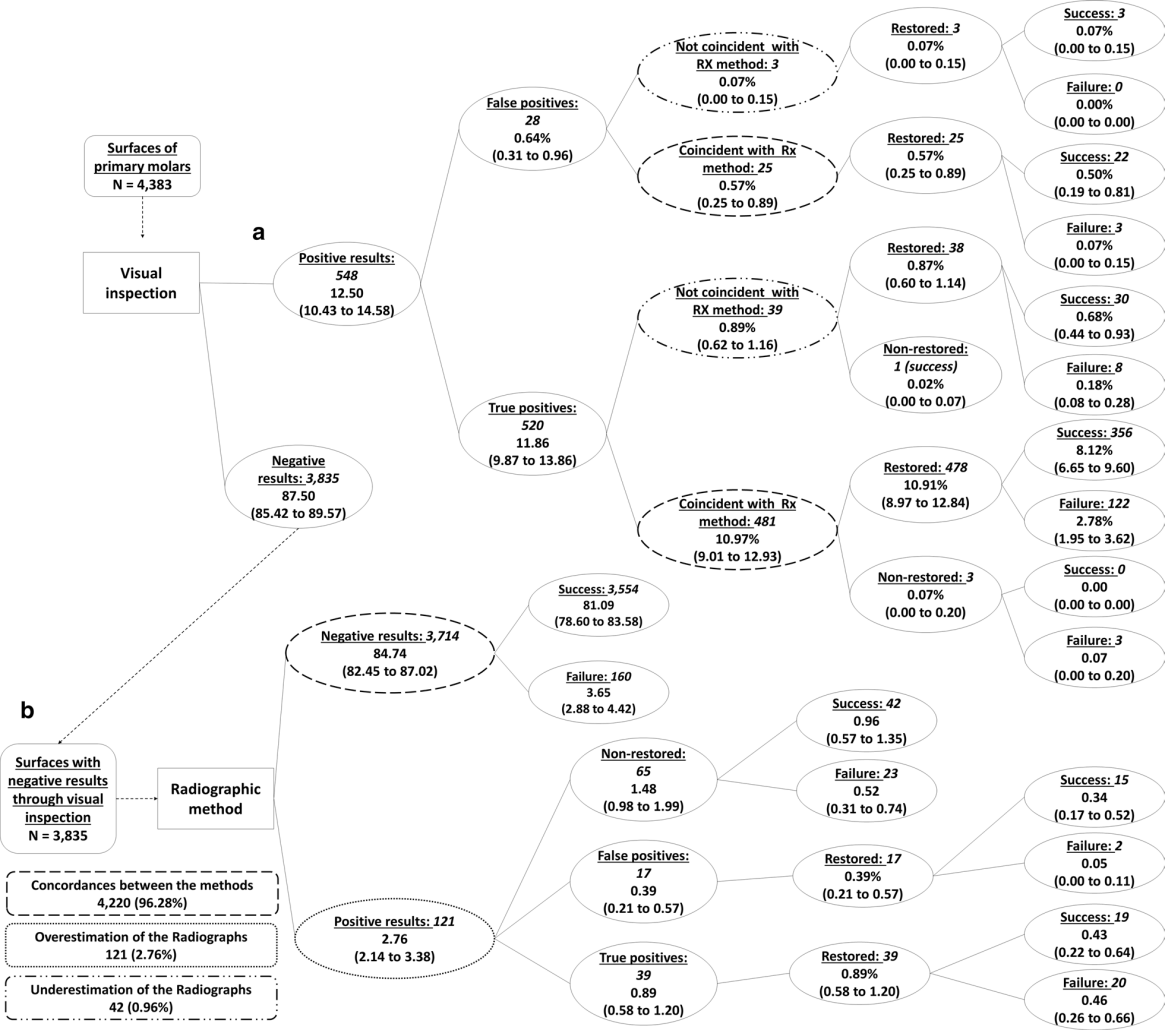
Decision tree for the decision of operative treatment for occlusal and proximal surfaces of primary molars decided first by visual inspection and then with adjunct radiographic method. Italicized numbers indicate the number of surfaces. Unformatted numbers indicated the frequency in relation to the total number of surfaces, and figures in parenthesis are the 95% confidence intervals adjusted by the cluster

The decision tree on Fig. 6 is related to the diagnosis made by different strategies in relation to the decision of operative treatment performed on occlusal and proximal surfaces of deciduous molars. This emphasis was given due to the assertion that radiographs are useful to detect caries lesions missed by visual inspection, which is the main reason to justify the use of the radiographs as a protocol for caries diagnosis in all children. Here, the vast majority of surfaces presented coincident results between both methods (more than 96%) (Fig. 6) when considering the indication for operative treatment in dental surfaces of deciduous molars. Discordances were observed in only 3.7% of the assessed surfaces. Radiographs indicated operative treatment more frequently than visual inspection in around 2.8% of cases (Fig. 6, pathway b). In around 1.0%, however, bitewings did not show radiolucency in surfaces classified as decayed visually (Fig. 6, pathway a).

For the results that would be reached with the use of the simultaneous association of methods, from non-operative with visual inspection to operative treatment with the radiographic method, changes in treatment decision would occur only in 121 surfaces (Fig. 6, pathway b). From these, 65 were not restored, and 23 failed (35.4%). Other 56 surfaces were restored, and 22 restorations (39.3%) needed to be replaced during the follow-up. No significant differences were observed when comparing these frequencies (OR = 1.10; 95%CI = 0.12 to 10.43).

On the other hand, if the results from both diagnostic methods had been considered as a sequential association, where bitewings would be used to confirm a positive result obtained by visual inspection, 42 dental surfaces (39 in the true-positive branch and 3 in the false-positive branch) would not have been indicated for operative treatment due to a negative result on the radiographic assessment. From these, 41 surfaces were restored and the failure rate was 19.5% (8 replaced restorations during the follow-up) (Fig. 6, pathway a).

### Occurrence of false-positive results

Figure 6 also shows the occurrence of false-positive results obtained by the use of both caries detection methods. A false-positive result was recorded when a dental surface was submitted to operative treatment, but when the absence of carious soft dentin after opening was observed. Consequently, all surfaces classified as false-positive results were restored.

We observed a total of 45 surfaces with false-positive results (1.02% considering all included surfaces). From these surfaces, 25 (55.6% of all false-positives) were diagnosed as positive for both methods (Fig. 6, pathway a). In 3 surfaces (6.7% from the false-positives), the decision for operative treatment was reached only by visual inspection (Fig. 6, pathway a), and in 17 surfaces (37.8%) the result was positive only with radiographic method (Fig. 6, pathway b).

### Evidence of overdiagnosis

The occurrence of overdiagnosis was estimated by assessing surfaces indicated for operative treatment by both detection methods, that were not restored and did not progress during the follow-up. Since the main clinical trial was designed to compare the simultaneous association of visual inspection with radiographic examination versus the visual inspection performed alone, only 4 dental surfaces, positively diagnosed at this threshold by visual inspection, were not restored due to issues in following the outlined treatment plan. Three of these surfaces progressed in the subsequent two years (Fig. 6, pathway a), indicating a low probability of overdiagnosis with visual inspection.

On the other hand, 65 dental surfaces indicated for operative treatment by the radiographic assessment were not restored (Fig. 6, pathway b). From these, 42 surfaces (64.6%) did not require operative interventions during the follow-up (Fig. 6, pathway b), which can be seen as an estimative of the radiographic method´s overdiagnosis on dental surfaces of deciduous molars with non-obvious clinical signs of caries lesions.

### Factors associated with the necessity of new operative interventions during the follow-up

According to the multilevel logistic regression analysis, when caries lesions, indicated for operative interventions, were detected with the radiographic method and missed by visual inspection, the occurrence of failures during the follow-up was significantly higher than when detected by both methods (Table 1). On the other hand, when the same type of lesions was detected by visual inspection, but not confirmed by the radiographic assessment, the occurrence of new operative treatments during the follow-up was similar to that when detected by both methods (Table 1). Evidently, when both methods were coincident in classifying a dental surface as sound (no intervention needed), the occurrence of new treatments was significantly lower in comparison to that when both methods detected lesions requiring restorations. These trends were observed in the univariate analysis, as well as in the multiple analysis adjusted by possible confounding variables. Moreover, when a possible mediator in the multiple model was added, same trend was observed (Table 1).

**Table 1:** Multilevel logistic regression considering the results from different diagnostic strategies for decision to restore the dental surfaces and occurrence of a new operative intervention during the follow-up

Explanatory variables	Univariate analyses	Multiple model 1**	Multiple model 2***
	Unadjusted OR (95%CI)	Adjusted OR (95%CI)	Adjusted OR (95%CI)
*Diagnostic results*			
Vis: positive; Rad: positive	1.00	1.00	1.00
Vis: positive; Rad: negative	0.78 (0.20–3.04)	0.85 (0.21–3.36)	0.85 (0.21–3.39)
Vis: negative; Rad: positive	4.52* (1.95–10.49)	6.12* (2.54–14.77)	8.62* (2.72–27.33)
Vis: negative; Rad: negative	0.09* (0.05–0.14)	0.13* (0.07–0.21)	0.23* (0.06–0.97)
*Treatment performed*			
Non-restored	1.00		1.00
Restored	11.43* (7.28–17.92)		1.87 (0.49–7.16)
*Dental surface*			
Proximal	1.00	1.00	1.00
Occlusal	1.91* (1.41–2.59)	1.28 (0.90–1.83)	1.29 (0.90–1.84)
*Type of tooth*			
1st molar	1.00	1.00	1.00
2nd molar	0.52* (0.33–0.81)	0.55* (0.34–0.90)	0.55* (0.34–0.90)
*Caries experience*			
dmf-s = 0–3	1.00	1.00	1.00
dmf-s > 3	8.71* (4.20–18.06)	5.35* (2.50–11.44)	5.37* (2.50–11.54)
*Child’s age*			
3 or 4 years old	1.00	1.00	1.00
5 or 6 years old	0.69 (0.33–1.43)	0.43* (0.21–0.85)	0.42* (0.21–0.85)

OR, odds ratio; 95%CI, 95% confidence intervals; Vis, visual inspection method; Rad, radiographic method; dmf-s, number of dental surfaces from primary teeth decayed, missed or filled

^*^Association statistically significant (p < 0.05)

^**^Multiple model 1 included only the confounding variables according to our predetermined conceptual framework

^***^Multiple model 2 included confounding variables and treatment performed as a possible mediator for failure occurrences

**Fig. 7:**
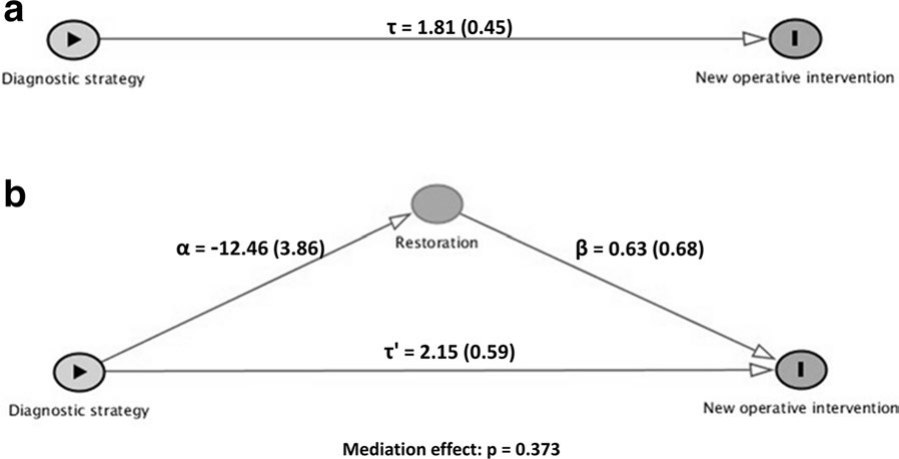
Mediation analysis to evaluate if performing a restoration mediates the occurrence of new operative interventions in the surfaces where caries lesions were missed by visual inspection but detected by radiographs (a: direct effect; b: mediated effect). Numbers represent the multilevel logistic regression coefficients (standard errors) adjusted by type of teeth, dental surface, caries experience and child’s age. *P* value was derived through Sobel test

A higher frequency of new operative interventions would be expected for cases where caries lesions, supposedly requiring operative treatment, were not detected by visual inspection but presented radiolucency reaching the dentin on radiographs. Part of these findings could have been related to defective restorations, and/or the incidence of new caries lesions missed at visual inspection. Therefore, and to evaluate if a restoration performed at baseline could exert any influence on the occurrence of new interventions during the follow-up in such cases, a mediation analysis was conducted. A significant and direct effect between the result obtained by the diagnostic strategy and the occurrence of new operative interventions was observed (Fig. 7a). This effect, however, remained significant after the inclusion of the mediator in the model, and the Sobel test indicated that the mediation effect of performing a restoration at the baseline was not statistically significant (Fig. 7b). This fact was probably due to the similar failure rates found for non-restored and restored surfaces: 35.4% of non-restored surfaces needed a restoration during the follow-up, and 39.3% of restorations needed to be replaced, respectively.

### Evidence of lead-time bias

**Fig. 8:**
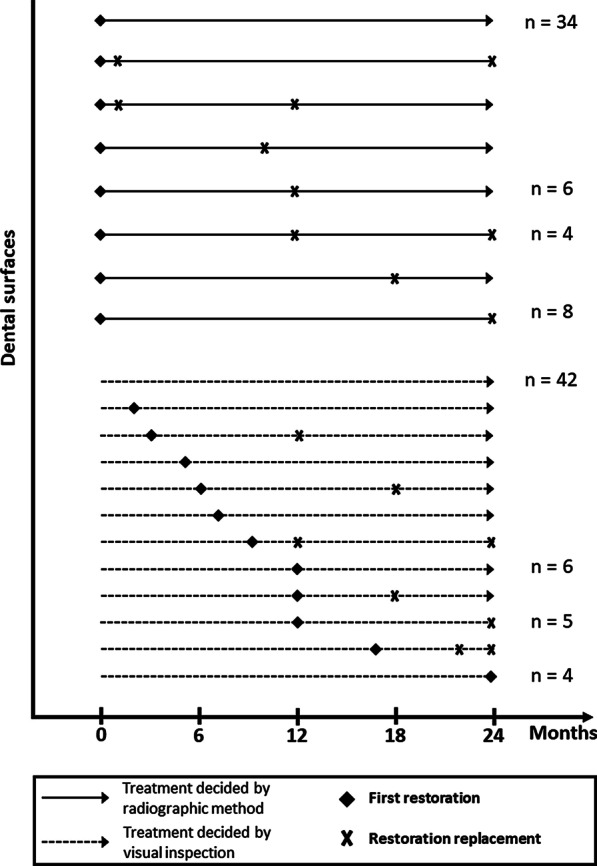
Time-to-event analysis for the surfaces with caries lesions detected only by radiographic method considering their clinical course (n = 121). Each arrow indicates a dental surface with a negative result through visual inspection, but positive result with radiographs. When N is indicated, there were more than one surfaces following the same clinical course

Despite the similar failure rates when comparing non-restored and restored dental surfaces with caries lesions detected only by radiographic assessment, the method could indicate the occurrence of lead time bias. To test such occurrence, a survival analysis using Cox regression for multiple-failure-time was conducted considering the 121 surfaces with caries lesions detected only by the radiographic method. When t0 was set at children´s birth date, a higher probability of failures for dental surfaces restored at the beginning of the study was observed (HR = 9.92; 95% CI = 5.78 to 17.02, *p* < 0.001). This trend can be clearly observed in Fig. 8. Thirty-four surfaces, restored at the beginning did not present failures throughout the study. However, other 18, also restored at the beginning of the study failed 22 times within the follow-up period. In relation to non-restored surfaces at the beginning of the study, 42 surfaces remained with no obvious cavities after 24 months (overdiagnosis made by the radiographic method). In addition, other 23 restored surfaces failed in 12 occasions (Fig. 8). This analysis reflects the occurrence of lead-time bias when therapeutic decisions are taken based on the simultaneous association of both caries detection strategies.

### When radiographs brought real benefits for caries diagnosis in preschool children

Despite the previously described issues, the diagnosis process made by associating visual inspection and radiographic findings could have presented some benefits.

Eight teeth were submitted to endodontic treatment during the follow-up. From these, in 5 teeth, caries lesions requiring operative intervention were detected by both methods and consequently restored at the baseline. One tooth was restored based on the radiographic assessment, this restoration failed and the tooth was subsequently submitted to endodontic treatment. The remaining two teeth presented caries progression reaching the pulp and needed endodontic treatment. In both cases, the caries lesions on proximal surfaces were overlooked by visual inspection, but the radiolucency was radiographically present. Therefore, these two teeth (corresponding to 6 dental surfaces) would benefit from the diagnosis made by the simultaneous association of both methods: visual and radiographic.

Moreover, 10 teeth were extracted due to caries related reasons. Two of them were extracted as a consequence of a failed endodontic treatment (already considered in the previous paragraph). Five were restored as indicated by both methods. For the remaining three, the presence of caries lesions was not observed by both methods. Thus, the radiographic method would not have a therapeutic impact compared to the visual inspection performed alone.

The radiographic method would also be beneficial for caries diagnosis in preschool children if the visual inspection presented false-positive results not confirmed by radiographs. This situation actually occurred in 3 surfaces (Fig. 6, pathway a). In other dental surfaces, an operative treatment decision was reached following a positive result (true positive) clinically observed but absent in the radiographic assessment. Eight of these surfaces required restoration replacements within the follow-up period (Fig. 6, pathway a).

The real benefits of the radiographic method could be observed in 17 dental surfaces (0.39% of all 4,383 surfaces) when considering the abovementioned possible scenarios.

Other aspects could also be considered as advantages of the radiographic method, although such benefits are not too evident. In 23 dental surfaces, no operative treatment was decided after visual inspection, while the radiographs presented positive results (Fig. 6, pathway b). However, these surfaces presented new caries lesions within the follow-up (17 surfaces) or were submitted to endodontic treatment, as described previously (6 surfaces). The benefit in these 17 surfaces is not too clear because this occurrence could be characterized as lead-time bias, as stated before.

Another situation concerns the restored surfaces that presented a positive result with the radiographic method but a negative through visual inspection. In this sample, 19 surfaces with these characteristics did not failed. This is not a clear benefit since part of these lesions could be cases of overdiagnosis.

Therefore, considering an optimistic estimative of the benefits of the radiographs for caries detection in deciduous molars, a total of 53 (6 + 3 + 8 + 17 + 19) dental surfaces (1.21% of all surfaces examined) possibly would have benefited from the radiographic method used in association with the visual inspection.

Although proximal surfaces have been pointed out as the type of surface that would have more benefits with the use of radiographs, a similar trend was observed compared to the total sample. At proximal and occlusal surfaces, possible benefits of the radiographic method were also observed in 1.21% and 1.81% of these surfaces, respectively. The decision trees related to the operative treatment divided by proximal and occlusal surfaces are presented as supplemental material (Additional files 1 and 2: Appendixes A and B).

## Discussion

The current diagnostic strategy for caries detection in children recommended by clinical guidelines is the simultaneous association of a clinical examination with radiographic assessment [9, 17, 23–26]. Nevertheless, such recommendation is based on accuracy studies as an attempt to minimize the problem of the visual inspection´s low sensitivity [27] since by associating both methods there´s a tendency to increase sensitivity [26]. However, most of the so mentioned accuracy studies were conducted at a laboratory setting and/or present a high risk of selection bias [26, 27]. Previous accuracy studies performed in representative samples of children seeking dental treatment, our target population, have shown that the indication of radiographs as a protocol for caries detection in all patients are not too useful [28, 29].

In order to clarify this controversial issue, we designed a randomized clinical trial comparing two caries detection strategies: the combination of information obtained by visual inspection + radiographic assessment versus the information from visual inspection performed alone [34, 35]. No statistically significant differences were observed in relation to the primary outcome (i.e. number of new operative interventions). Moreover, children allocated to the RAD group received more restorations throughout the whole study and presented more false-positive results in comparison to children diagnosed and treated according to the visual inspection alone [35].

A secondary analysis, considering each dental surface as the unit of analysis, was here performed to evaluate the clinical course of all occlusal and proximal surfaces of deciduous molars that were diagnosed using both methods, but treated in accordance with the allocation group. This is the main strength of our study. By using this approach associated with the study design, it was possible to evaluate the therapeutic impact of the radiographic method on the visual inspection, and to compare the treatment success that was performed based on visual inspection alone or in combination with radiographic assessment. In addition, it was estimated the overdiagnosis (mainly for the radiographic method) as well as the lead-time bias possible occurrence.

With regard to the therapeutic impact, both diagnosis and treatment decisions made after the assessment of radiographs did not change the majority of diagnosis and treatment decisions made by visual inspection alone. When all types of treatments were considered (non-operative or operative), the diagnosis reached with the radiographic assessment would change the decision in less than 30% of surfaces. Moreover, most discrepancies (almost 25%) were seen for initial caries lesions, corroborating previous observations on how visual inspection is more accurate than radiographs at this threshold [26, 27], and how radiographs tend to underestimate this type of lesions [15, 43]. Therefore, in the simultaneous association of methods, the results obtained with radiographs would not change the classification made by visual inspection, contradicting authors who advocate the use of radiographs to detect caries lesions before cavitation, since most initial lesions were not observed on bitewings [9, 17, 24–26].

This argument, however, could be valid for non-cavitated lesions presenting radiolucency related to the enamel or the outer third of dentin. Less than 3% of all surfaces were classified as sound by visual inspection but presented radiolucency on radiographs. The success of non-operative interventions placed on these surfaces based on the radiographic results was not superior than that of surfaces that did not receive any treatment whatsoever. Therefore, the use of radiographs for early detection of caries lesions before cavitation did not present any advantages against the visual inspection performed alone.

One of the motives to incorporate radiographs as a protocol for caries detection in children is related to the capability of the method to detect more advanced lesions that could be overlooked during clinical examination. The decision of operative intervention on proximal and occlusal surfaces of deciduous molars was discordant in less than 4% of surfaces between methods. When the simultaneous association of methods was considered, differences were observed in 2.7% of the surfaces. Contrariwise, when radiographs were used to confirm a positive result obtained with visual inspection (sequential association), a therapeutic change occurred only in about 1% of all surfaces. Hence, a negligible therapeutic impact of the radiographs on the decision made using visual inspection alone was observed.

Taking into account the principle of parsimony, the last finding would be sufficient not to recommend radiographs as a diagnostic strategy for caries detection in children. In addition, we must consider the cumulative hazards of ionizing radiation in children since they are more sensitive to its effects than adults [44]. Another point of concern is related to costs. The economic aspects related to this study will be presented in a future manuscript.

The occurrence of false-positives was an expected harm confirmed in our study [45]. The radiographic method and its simultaneous association with visual inspection have presented lower specificity than the visual inspection performed alone in several accuracy studies [26, 28, 29, 31]. Although most false-positive results were coincident between methods, a higher number of dental surfaces presented false-positive exclusively with the radiographic method (17 surfaces) in comparison to visual inspection alone (3 surfaces).

Another strength of our study is that, for the first time, the overdiagnosis with the use of the radiographic method for caries lesions detection was appraised. This was possible since some clinically sound surfaces, but presenting radiolucency reaching the dentin, were not treated operatively. Around 65% of these, were overdiagnosed, at least considering a period of two years.

These lesions were not restored since the decision was made only by visual inspection. However, they could progress, and a restoration would be necessary to be placed afterwards. On the other hand, if these surfaces had a radiolucency reaching the dentin, and the treatment had been based on the simultaneous association of visual and radiographic methods, the restorations would be performed at the beginning of the study, and these restorations could have failed during the follow-up.

To evaluate if the anticipated operative treatment could have reduced the failure due to avoiding the lesion progression, we conducted a mediation analysis. However, failure rates were similar between the restored or not restored surfaces. Therefore, performing the restoration at the beginning of the study did not influence the failure rate of the dental surfaces when this combination of results occurred (negative in the visual inspection but positive through the radiographic evaluation).

On the other hand, a possible lead-time bias could occur in the dental surfaces restored earlier because of the result obtained with the radiographic method [45]. When the survival analysis for multiple failures was conducted, with the first restoration as the first event and necessity of restoration replacement as further events, it was observed that performing a restoration due to a positive result obtained with the radiographic method is a clear example of lead-time bias. This situation would only be beneficial if the failure rate of restorations was lower than the occurrence of new lesions in non-restored surfaces. However, the frequency of failures in the restorations made at the baseline was similar to the frequency of surfaces with new caries lesions during the follow-up. Therefore, the simultaneous association of visual inspection and radiographic assessment unnecessarily anticipated the operative treatment, characterizing the occurrence of lead-time bias.

In very few cases, however, the results obtained with the radiographic assessment presented benefits. In 143 dental surfaces presenting discordant results between methods at the decision for operative treatment, we estimated that 53 surfaces were possibly benefited from the radiographic method. Nonetheless, in view of the several reported possibilities of harms, the low number of dental surfaces that could receive a benefit from radiographs does not justify the incorporation of the method for caries detection of children as a regular clinical routine.

Findings of this study should be interpreted cautiously. Our examiners and care providers were experienced and well-trained clinicians. They were trained before the study and reached high values of reliability. Moreover, the study was conducted following the well-controlled characteristics of clinical trials. Multicenter trials and further pragmatic studies should be carried out to increase the external validity of the present results. Observations made in radiographs taken in children for other reasons and the natural clinical course of eventual radiolucencies could give additional evidence to corroborate our results. Besides, our findings can be extrapolated only for caries detection in deciduous teeth. The performance of diagnostic strategies for caries detection considering oral health outcomes for patients in other age groups should be further tested in longitudinal studies.

## Conclusions

In conclusion, the use of radiographs in the diagnostic strategy for caries detection in children brings more harms than benefits. Reasons rely on the low therapeutic impact as well as the occurrence of false-positive results, overdiagnosis and lead-time bias. Visual inspection brings more benefits considering the clinical course of dental surfaces of deciduous molars. Clinical guidelines related to caries care [9, 17, 23, 24] should revise the current recommendations around caries diagnosis in children.

## Supplementary Information

**Additional file 1.** Decision tree related to the decision for operative treatment in proximal surfaces. (TIF 1827 KB)

**Additional file 2.** Decision tree related to the decision for operative treatment in occlusal surfaces. (TIF 1810 KB)

## Data Availability

The raw trial data after de-identification is shared at the public repository of the University of São Paulo (USP), http://repositorio.uspdigital.usp.br/handle/item/259. The data management plan can be accessed at https://dmptool.org/plans/55407/export.pdf?export%5Bquestion_headings%5D=true. The lead author (the manuscript’s guarantor) affirms that the manuscript is an honest, accurate, and transparent account of the study being reported; that no important aspects of the study have been omitted; and that any discrepancies from the study as planned and registered have been explained.

## Abbreviations

CARDEC: The CARies DEtection in Children
VIS: Visual inspection
RAD: Radiographic method
ICDAS: International Caries Detection and Assessment System
95%CI: 95% Confidence interval
OR: Odds ratio
HR: Hazard ratio
