# Supplementary figures and images for: Negligible therapeutic impact, false-positives, overdiagnosis and lead-time are the reasons why radiographs bring more harm than benefits in the caries diagnosis of preschool children
Source: BMC Oral Health. 2021 Mar 31;21:168. doi: 10.1186/s12903-021-01528-w (PMC8011211; doi:10.1186/s12903-021-01528-w)

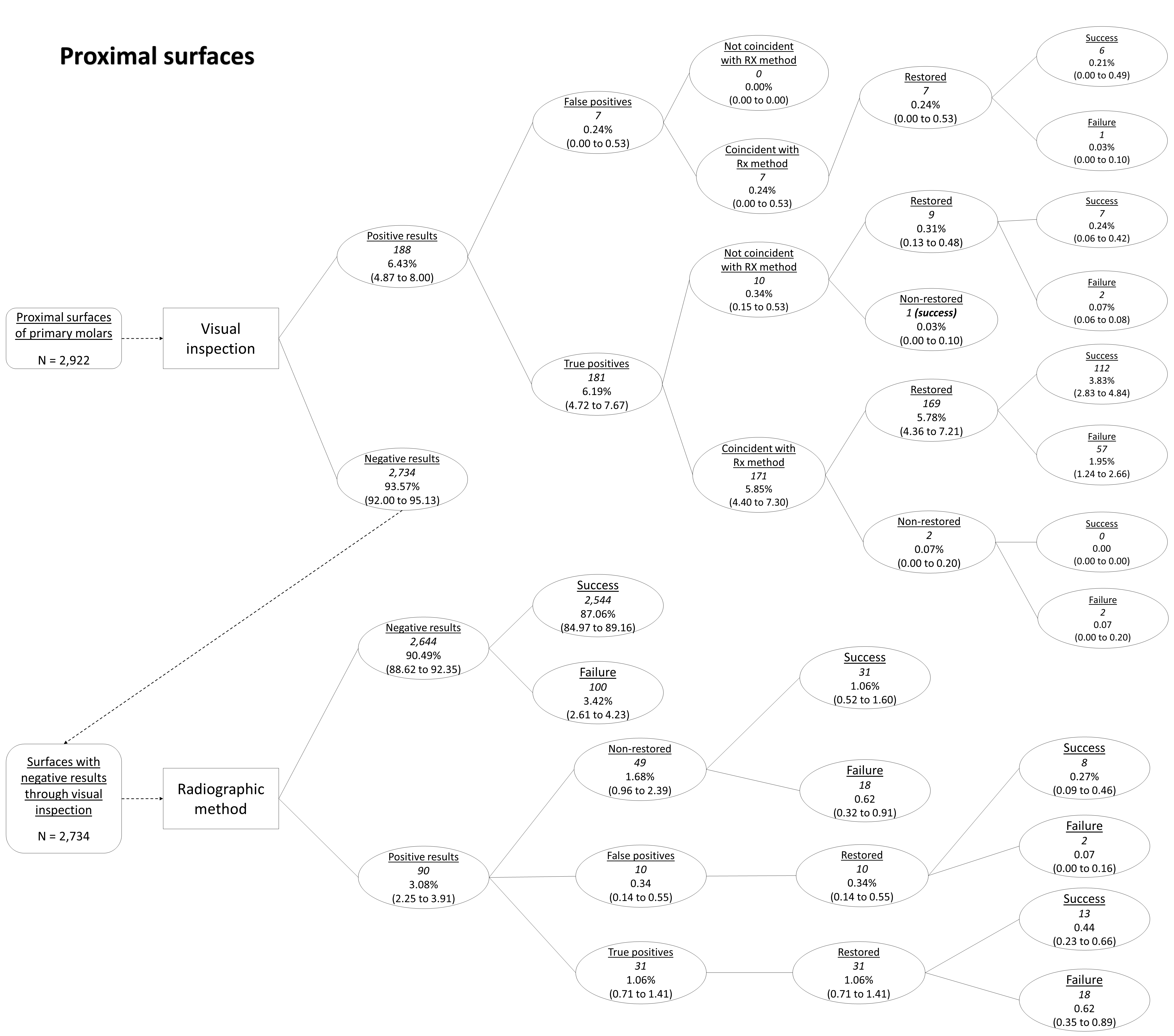

Supplement: Supplementary file 1 — Additional file 1. Decision tree related to the decision for operative treatment in proximal surfaces. (TIF 1827 KB) [file 12903_2021_1528_MOESM1_ESM.tif]

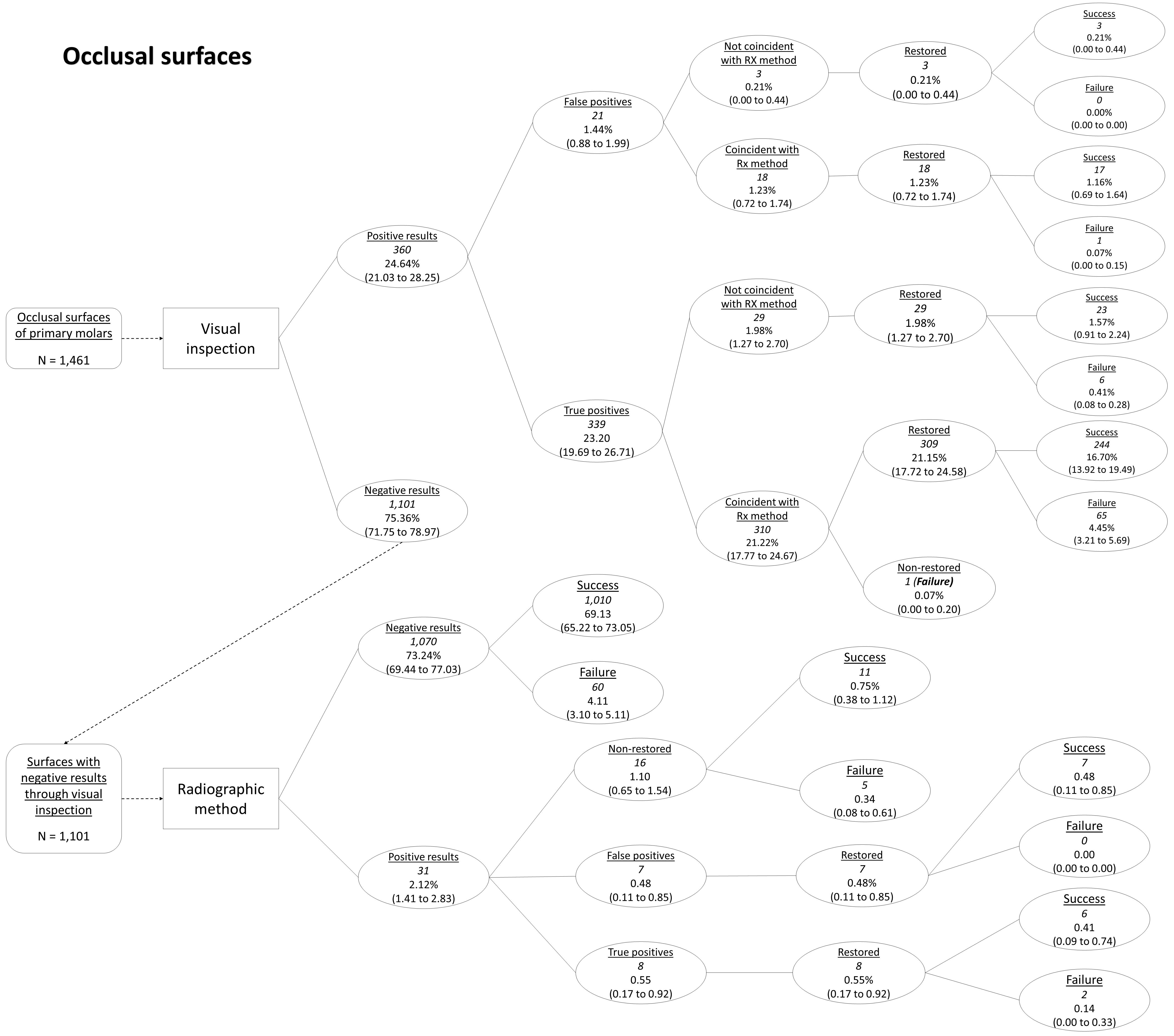

Supplement: Supplementary file 2 — Additional file 2. Decision tree related to the decision for operative treatment in occlusal surfaces. (TIF 1810 KB) [file 12903_2021_1528_MOESM2_ESM.tif]
